# Primary site surgery of de novo stage IV HER2-positive breast cancer in the era of new drug treatments

**DOI:** 10.3389/fonc.2023.1308854

**Published:** 2024-01-09

**Authors:** Guanyu Lu, Lin Jia, Ruohan Yang, Yutong Liu, Zheng Lv, Jiuwei Cui

**Affiliations:** ^1^ Cancer Center, The First Hospital of Jilin University, Changchun, Jilin, China; ^2^ Departments of Breast Surgery, The First Hospital of Jilin University, Changchun, Jilin, China

**Keywords:** stage IV, breast cancer, HER2-positive, surgery, prognosis

## Abstract

**Objective:**

The surgical treatment of the primary site has been a subject of controversy in patients with de novo metastatic breast cancer. In recent years, studies using large databases and retrospective analyses have provided evidence of the survival benefits of localized surgery for these patients. However, due to the improved prognosis associated with novel antitumor agents and the widespread use of anti-HER2 therapy, it is important to investigate the role of primary site surgery in the context of new drug treatments for stage IV HER2-positive breast cancer.

**Methods:**

This retrospective analysis included patients with metastatic breast cancer at diagnosis who were consulted at the First Hospital of Jilin University between 2016 and 2022. We compared the patients’ clinical and pathological characteristics, treatment regimens, and prognosis between the surgery and non-surgery groups.

**Results:**

A total of 96 patients with stage IV HER2-positive breast cancer were included in the study, with 24 patients (25%) undergoing surgery for the primary lesion. Patients with lower Eastern Cooperative Oncology Group (ECOG) scores, earlier T-stage, metastases confined to one organ/site, and fewer metastases were more likely to undergo surgery. Patients in the surgical group had longer progression-free survival (median 25.7 *vs*. 15.9 months, p=0.073) and overall survival (median 79.1 *vs*. 48 months, p=0.073) compared to patients in the non-surgical group, however, there was no statistical difference. Univariate and multivariate Cox regression analysis suggested that the choice of first-line targeted therapy regimens rather than surgical treatment influenced the patients’ prognoses. In the subgroup of patients receiving first-line targeted therapy with trastuzumab plus pertuzumab, the decision to undergo surgery on the primary site did not have a statistically significant effect on prognosis.

**Conclusion:**

Primary site surgery does not improve the prognosis of de novo stage IV HER2-positive breast cancer. In the era of anti-HER2 therapy, primary surgery is not recommended, except in exceptional circumstances.

## 1 Introduction

Breast cancer is currently the most common type of malignancy worldwide, with distant metastases observed in 5-10% of patients upon initial diagnosis (1, 2). For metastatic disease, the primary objective of current treatment approaches is to slow down disease progression and extend survival time, with systemic therapy serving as the foundation of the treatment plan (3). However, around 30%-50% of patients undergo localized treatment, primarily through surgery (4). Presently, it is believed that local surgical intervention can reduce local symptoms such as pain, skin ulcers, and bleeding. Additionally, combined with systemic therapy, localized treatment can also potentially reduce tumor burden and minimize the risk of tumor metastasis (5). Nevertheless, the significance of localized therapy in stage IV breast cancer remains uncertain (6).

While there are currently no specific guidelines for the local treatment of de novo metastatic breast cancer patients, four prospective clinical trials provide valuable insights. The TBCRC013 study and the MF07-01 study included patients with advanced disease who were initially treated and randomized into two groups: one receiving systemic therapy after surgery, and the other receiving systemic therapy alone. On the other hand, the TATA study and the EA2108 study enrolled advanced patients who had responded well to systemic therapy and randomized them into a surgical group and a nonsurgical group. The MF07-01 study showed that patients who received localized treatment experienced improved survival (7, 8). But there was also selection bias in this trial, and the majority of patients in the surgery group were hormone receptor (HR) positive patients, which may have a better prognosis. However, the TATA study, TBCRC013 study, and EA2108 phase III clinical trial suggested that localized surgical management does not enhance survival in patients with stage IV breast cancer (9–11).

HER2-positive breast cancer, a subtype that once had a poor prognosis, has achieved remarkable outcomes with systemic therapy in the era of targeted therapies, significantly improving the prognosis of patients. Therefore, the question of whether surgical treatment of the primary breast lesion in HER2-positive patients with de novo advanced breast cancer would provide additional benefits to this subset of patients is commonly encountered in clinical practice. Several retrospective studies have suggested that patients with de novo metastatic HER2-positive breast cancer can benefit from localized treatment of the primary site (6, 12, 13). However, not all of the patients included in these previous studies received anti-HER2 therapy, which fails to address the current clinical question at hand. Therefore, we conducted a retrospective analysis utilizing the most recent clinical data in order to investigate the clinical characteristics and prognosis of patients with de novo stage IV HER2-positive breast cancer, with or without primary site surgery. Our objective was to figure out which patients would benefit from local treatment.

## 2 Methods

### 2.1 Study population

Here we included patients initially diagnosed with stage IV HER2 positive breast cancer at the Cancer Center of the First Hospital of Jilin University between January 2016 to December 2022. The inclusion criteria in this study are as follows: 1) Pathologically confirmed as invasive breast carcinoma; 2) Initial diagnosis of metastatic breast cancer; 3) Complete clinical data and pathology of the patients; 4) All patients received anti-HER2 targeted therapy.

The patients with concomitant other primary tumors and metastatic cancer of unknown primary sites were excluded. Our retrospective study did not involve any intervention factors, physical or economic burden, or adverse effects on the patients. Personal information will be kept confidential in all clinical data.

### 2.2 Data collection

Patient data were gathered by reviewing their medical records. The information extracted included the age at which the patients were diagnosed with breast cancer, their menstrual status, pathologic type of cancer, tumor stage, histological grade, hormone receptor status (estrogen and progesterone receptor), Ki-67 value, metastatic site and number of metastases, first-line treatments, time to disease progression, and time to death. Adverse events caused by surgery were also collected in the surgical group.

### 2.3 Statistical analysis

Continuous variables were shown as median values with range. Categorical variables were presented as frequencies with percentages. The chi-square test or Fisher’s exact test was used to compare the clinical characteristics of patients in the surgery and non-surgery groups. Survival statistics were assessed using Kaplan-Meier analysis, and the log-rank test was used to determine progression-free survival (PFS) and overall survival (OS) between the two groups. Univariate and multivariate Cox regression analyses were performed to estimate the impact of surgery on prognosis. Progression-free survival (PFS) was defined as the time from the first diagnosis of breast cancer to disease progression or death from any cause. Overall survival (OS) was defined as the time from the first diagnosis of breast cancer to death.

Statistical significance was defined as a two-sided p-value less than 0.05. The statistical tests were performed using SPSS 26.0 (SPSS Inc., Chicago, IL) and R software, version 4.2.2.

## 3 Results

### 3.1 Population characteristics

A total of 96 patients with stage IV HER2-positive breast cancer were included in our study. Out of these patients, 24 (25%) underwent surgery for the primary lesion. **Table 1** presents the characteristic differences between the two groups: the surgery group and the non-surgery group. Our findings indicate that patients who underwent surgery for the primary lesion had a higher likelihood of having a lower Eastern Cooperative Oncology Group (ECOG) score (p=0.004), the absence of lung metastasis (p=0.024) and visceral metastasis (p=0.030), only bone metastasis (p=0.010), metastases confined to one organ/site (p=0.002), and a lower number of metastases (p<0.001). There were no significant differences in other characteristics such as age distribution, menstrual status, pathological type, T stage, N stage, histological grading, ER/PR status, Ki67 value, prevalence of bone and liver metastasis, and first-line target therapy.

**Table 1:** clinicopathological characteristics of patients in surgery and non-surgery groups.

Items	Surgery	Non-Surgery	*p*
	n=24 (%)	n=72 (%)	
Age			
<50	13 (54.2%)	33 (45.8%)	0.479
≥50	11 (45.8%)	39 (54.2%)	
Menstrual status			
Premenopausal	13 (54.2%)	38 (52.8%)	0.906
Postmenopause	11 (45.8%)	34 (47.2%)	
Pathological type			
Invasive ductal carcinoma	24 (100%)	68 (94.4%)	0.677
Invasive lobular carcinoma	0 (0%)	3 (4.2%)	
Unknown	0 (0%)	1 (1.4%)	
ECOG scores			
0	22 (91.7%)	39 (54.2%)	0.004
1	2 (8.3%)	32 (44.4%)	
2	0 (0%)	1 (1.4%)	
T stage			
T0-2	18 (75.0%)	39 (54.2%)	0.072
T3-4	6 (25.0%)	33 (45.8%)	
N stage			
N0-1	10 (41.7%)	36 (50.0%)	0.479
N2-3	14 (58.3%)	36 (50.0%)	
Histological grading			
1	0 (0%)	0 (0%)	0.319
2	10 (41.7%)	25 (34.7%)	
3	8 (33.3%)	17 (23.6%)	
Unknown	6 (25.0%)	30 (41.7%)	
ER status			
Negative	16 (66.7%)	43 (59.7%)	0.545
Positive	8 (33.3%)	29 (40.3%)	
PR status			
Negative	17 (70.8%)	44 (61.1%)	0.391
Positive	7 (29.2%)	28 (38.9%)	
Ki67 value			
<15%	2 (8.3%)	4 (5.6%)	0.638
≥15%	22 (91.7%)	68 (94.4%)	
Metastatic site			
Bone			
No	9 (37.5%)	28 (38.9%)	0.904
Yes	15 (62.5%)	44 (61.1%)	
Lung			
No	21 (87.5%)	45 (62.5%)	0.024
Yes	3 (12.5%)	27 (37.5%)	
Liver			
No	17 (70.8%)	42 (58.3%)	0.276
Yes	7 (29.2%)	30 (41.7%)	
Only soft tissue			
No	22 (91.7%)	69 (95.8%)	0.596
Yes	2 (8.3%)	3 (4.2%)	
Only bone			
No	12 (50.0%)	56 (77.8%)	0.010
Yes	12 (50.0%)	16 (22.2%)	
Confined to one organ/site			
No	4 (16.7%)	38 (52.8%)	0.002
Yes	20 (83.3%)	34 (47.2%)	
Visceral metastasis			
No	14 (58.3%)	24 (33.3%)	0.030
Yes	10 (41.7%)	48 (66.7%)	
Number of metastasis			
1	12 (50.0%)	6 (8.3%)	<0.001
2-3	3 (12.5%)	6 (8.3%)	
≥4	9 (37.5%)	60 (83.3%)	
First line target therapy			
Trastuzumab	16 (66.7%)	35 (48.6%)	0.125
Trastuzumab+Pertuzumab	8 (33.3%)	37 (51.4%)	

### 3.2 Clinical characteristics of patients with surgery

A total of 24 patients underwent surgical treatment for breast lesions in this study, with 19 undergoing radical breast surgery (simple mastectomy on affected side and axillary lymph node dissection) and five patients undergoing palliative surgery (resection of mastectomy and axillary lymph node dissection if necessary). Six patients (25%) received surgical treatment before systemic therapy; three had oligometastases, and one had bone metastases only. Eighteen patients (75%) underwent surgery following systemic treatment, with ten patients having bone-only metastases and eight patients having oligometastases. Three patients had visceral (lung and liver) oligometastases, and the efficacy of systemic therapy was evaluated as partial response (PR) in 2 patients and complete response (CR) in 1 patient. Three patients had soft tissue-only metastases and underwent surgery after the complete response of the metastases. No serious adverse events were observed in any of the 24 patients who underwent surgery. None of the suspensions of targeted therapy due to surgery exceeded two months.

### 3.3 Survival outcome

**Figure 1:**
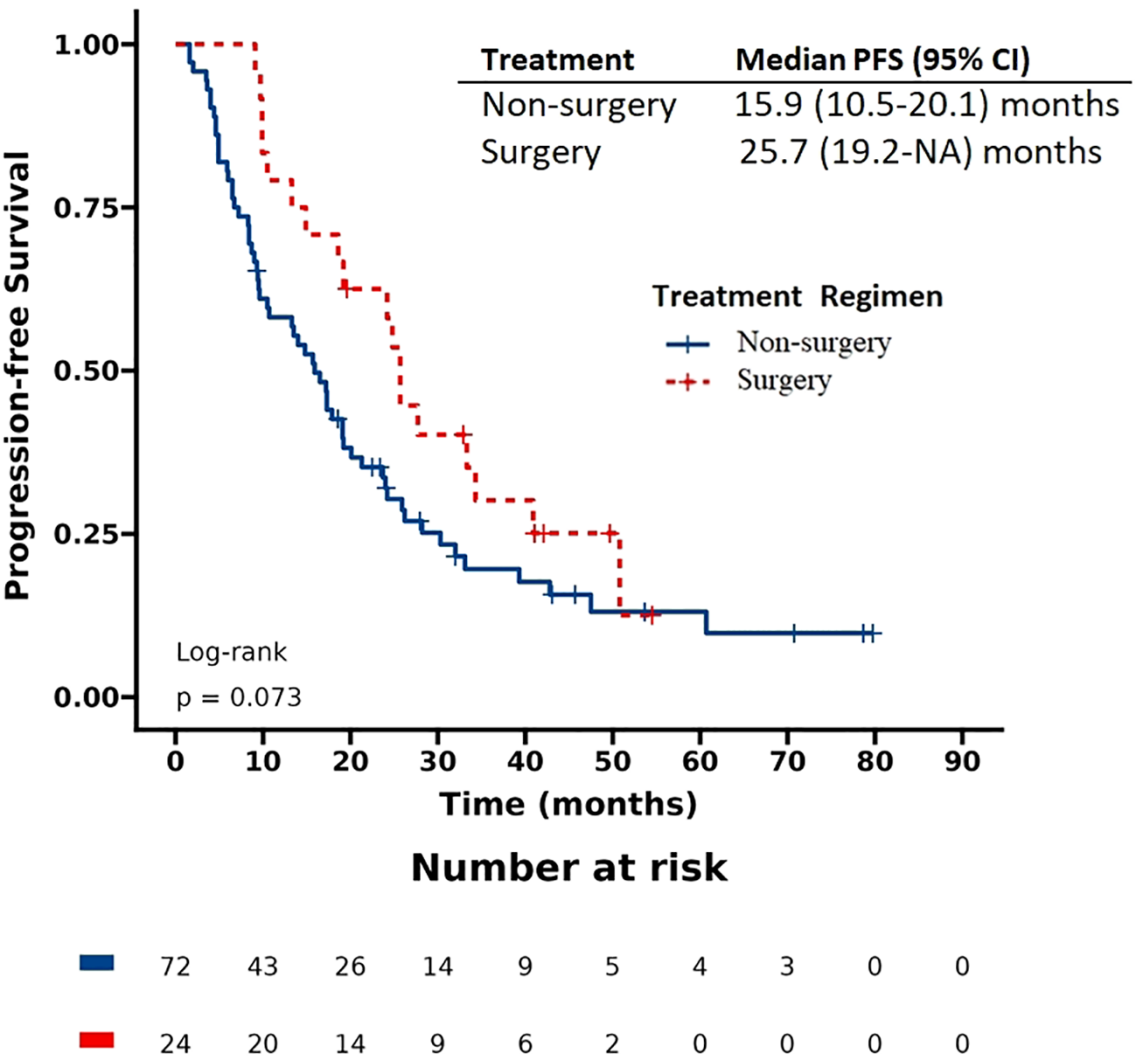
Progression-free survival (PFS) in patients with de novo stage IV HER2 positive breast cancer.

**Figure 2:**
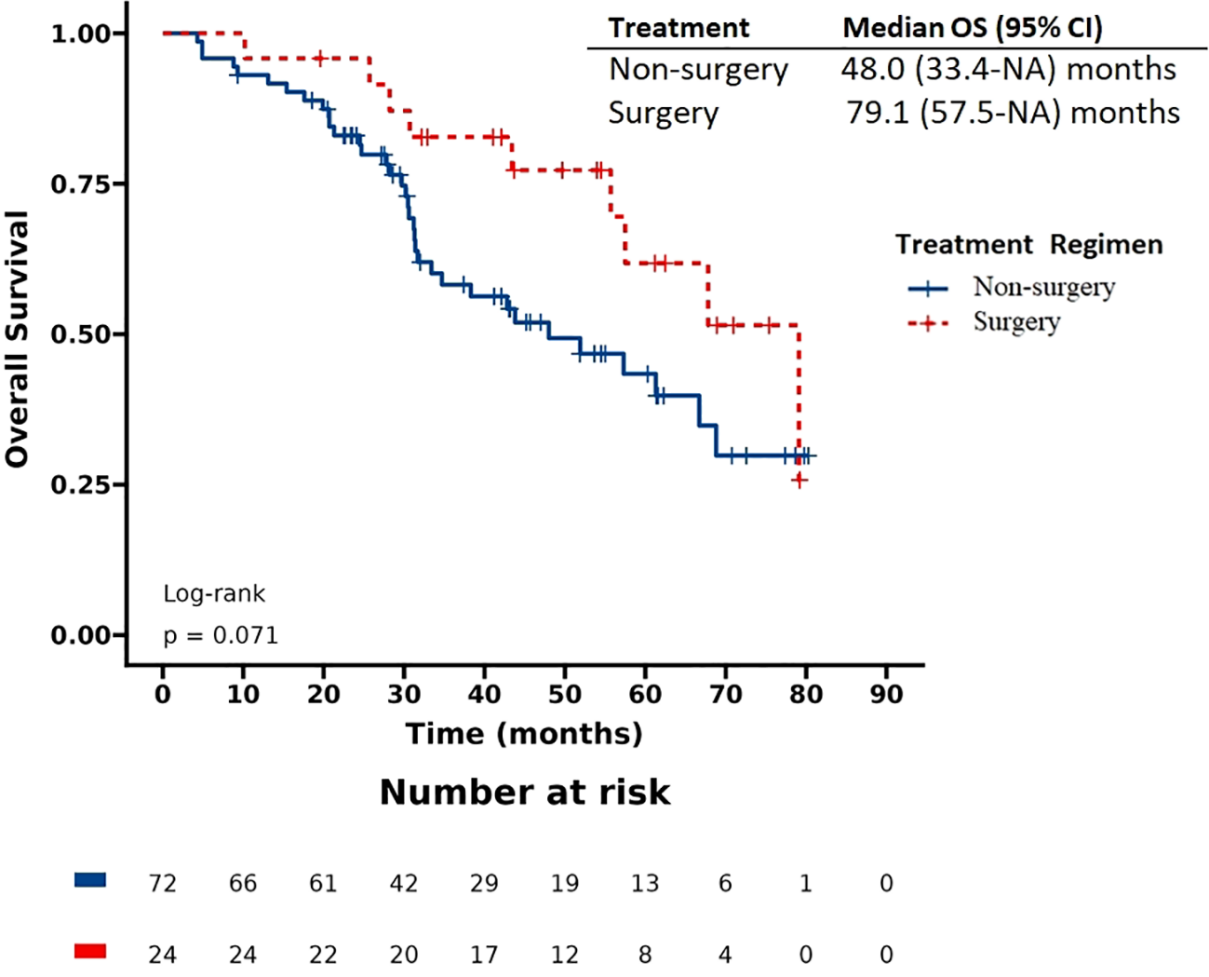
Overall survival (OS) in patients with de novo stage IV HER2 positive breast cancer.

The patients had a median progression-free survival (PFS) of 17.9 months and a median overall survival (OS) of 57.5 months. Based on the Kaplan-Meier survival curve presented in **Figures 1**, **2**, we observed no significant differences in PFS between the surgery and non-surgery groups at the primary site. The median PFS for the surgery group was 25.7 months (19.2 to NA months), while the non-surgery group had a median PFS of 15.9 months (10.5 to 20.1 months). Similarly, we found no significant difference in OS between the surgery and non-surgery groups. The median OS for the surgery group was 79.1 months (57.5 to NA months), while the non-surgery group had a median OS of 48 months (33.4 to NA months).

We utilized the Cox hazard model to investigate whether surgical treatment improved the prognosis of patients. Univariate analysis indicated that the following factors were significantly associated with progression-free survival (PFS): ECOG score, T stage, lung metastasis, liver metastasis, bone metastasis as the only site of metastasis, metastatic lesions confined to one organ/site, visceral metastasis, and receiving first-line target therapy (**Table 2**). Multivariable analysis demonstrated that first-line target therapy was an independent prognostic factor for PFS. However, there was no statistically significant difference in PFS improvement with surgery.

**Table 2:** Univariate and multivariate analysis of the prognostic factors for progression-free survival (PFS).

Items	Univariate		Multivariate	
	Hazard ratio value (95%CI)	*P*	Hazard ratio value (95%CI)	*P*
ECOG scores				
0	1.000		1.000	
1	2.678 (1.671-4.292)	<0.001	2.046 (0.993-4.218)	0.052
2	11.640 (1.486-91.160)	0.019	7.479 (0.782-71.504)	0.081
T				
T0-2	1.000		1.000	
T3-4	1.793 (1.127-2.855)	0.014	0.699 (0.340-1.438)	0.330
Lung metastasis				
No	1.000		1.000	
Yes	1.850 (1.147-2.985)	0.012	2.215 (1.045-4.694)	0.038
Liver metastasis				
No	1.000		1.000	
Yes	2.165 (1.350-3.472)	0.001	2.391 (0.950-6.019)	0.064
Only bone metastasis				
No	1.000		1.000	
Yes	0.555 (0.335-0.917)	0.022	0.945 (0.344-2.592)	0.912
Confined to one organ/site				
No	1.000		1.000	
Yes	0.607 (0.386-0.954)	0.030	0.876 (0.425-1.806)	0.720
Visceral metastasis				
No	1.000		1.000	
Yes	2.330 (1.434-3.785)	0.001	0.875 (0.255-3.006)	0.833
First line target therapy				
Trastuzumab	1.000			
Trastuzumab+Pertuzumab	0.483 (0.303-0.772)	0.002	0.385 (0.221-0.671)	0.001
Surgery				
No	1.000		1.000	
Yes	0.619 (0.364-1.052)	0.076	0.706 (0.387-1.288)	0.256

Similarly, we observed a significant association between the following factors and overall survival (OS): ECOG score, T stage, liver metastasis, bone metastasis as the only site of metastasis, metastatic lesions confined to one organ/site, visceral metastasis, receiving first-line target therapy, and undergoing surgery (**Table 3**). Multivariable analysis showed that first-line targeted therapy was the independent prognostic factor for overall survival. Similarly, there was no statistically significant difference in improved OS with surgery.

**Table 3:** Univariate and multivariate analysis of the prognostic factors for overall survival (OS).

Items	Univariate		Multivariate	
	Hazard ratio value (95%CI)	*P*	Hazard ratio value (95%CI)	*P*
ECOG scores				
0	1.000		1.000	
1	3.188 (1.701-5.975)	<0.001	1.538 (0.664-3.563)	0.315
2	84.361 (7.396-962.239)	<0.001	–	0.987
T				
T0-2	1.000		1.000	
T3-4	2.803 (1.533-5.126)	0.001	1.574 (0.718-3.451)	0.258
Liver metastasis				
No	1.000		1.000	
Yes	2.228 (1.222-4.060)	0.009	1.086 (0.442-2.673)	0.857
Only bone metastasis				
No	1.000		1.000	
Yes	0.235 (0.099-0.561)	0.001	0.391 (0.128-1.196)	0.100
Confined to one organ/site				
No	1.000		1.000	
Yes	0.434 (0.238-0.792)	0.006	0.589 (0.255-1.360)	0.215
Visceral metastasis				
No	1.000		1.000	
Yes	2.670 (1.366-5.218)	0.004	0.784 (0.276-2.230)	0.648
First line target therapy				
Trastuzumab	1.000			
Trastuzumab+Pertuzumab	0.491 (0.257-0.940)	0.032	0.434 (0.207-0.908)	0.027
Surgery				
No	1.000		1.000	
Yes	0.514 (0.246-1.074)	0.077	0.627 (0.272-1.442)	0.272

### 3.4 Subgroup analysis

**Figure 3:**
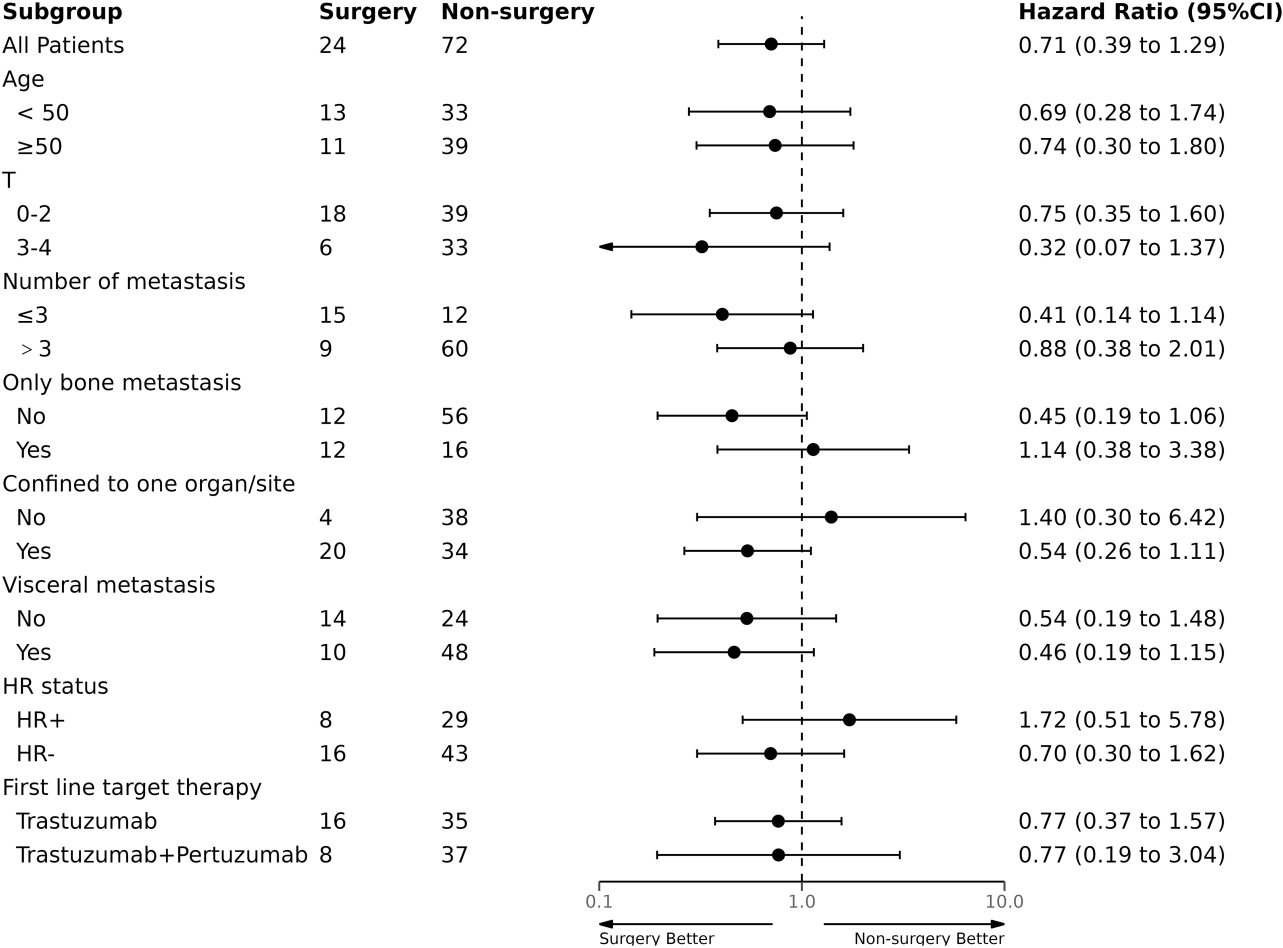
Subgroup analysis of Progression-free survival (PFS).

**Figure 4:**
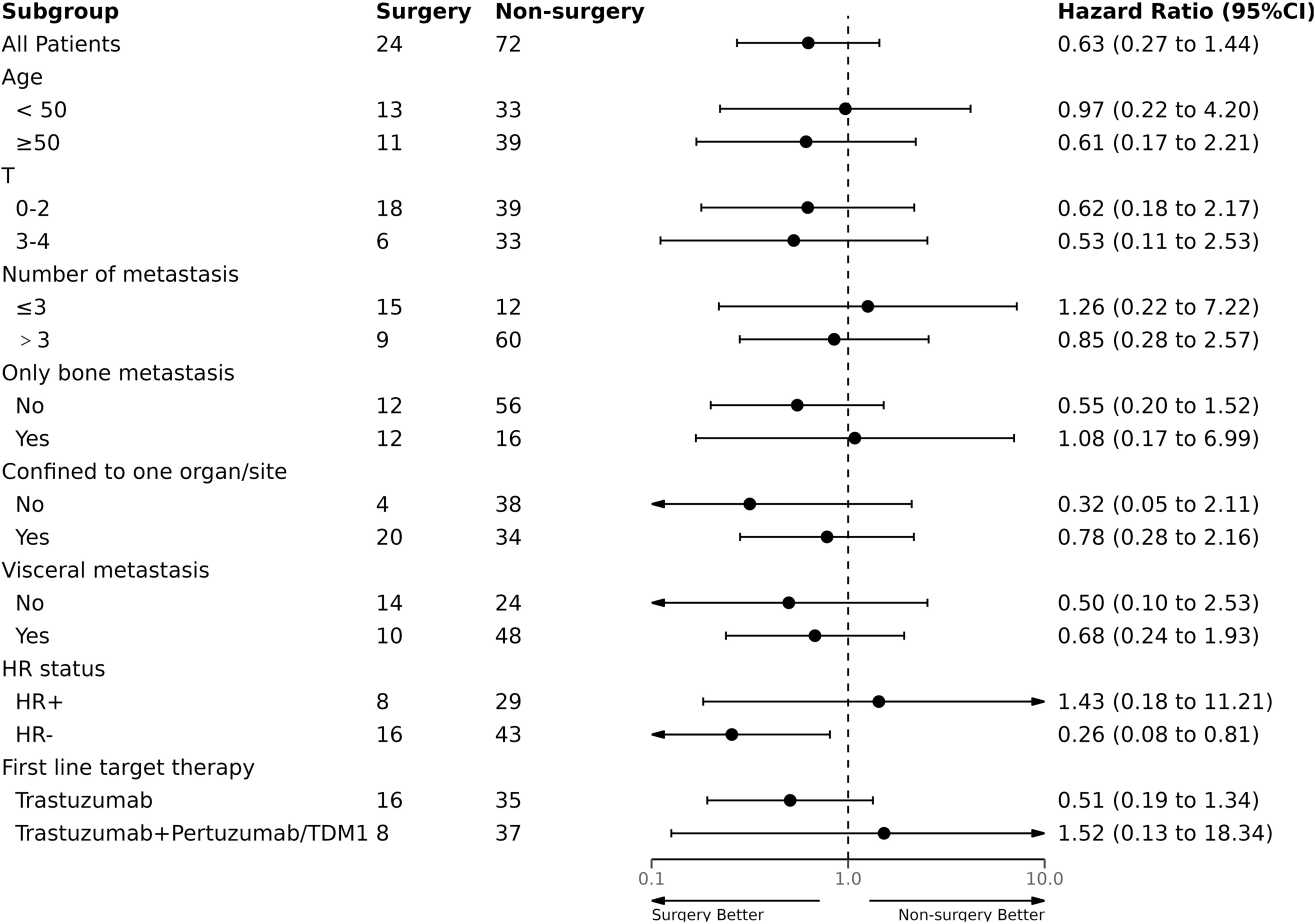
Subgroup analysis of overall survival (OS).

**Figure 5:**
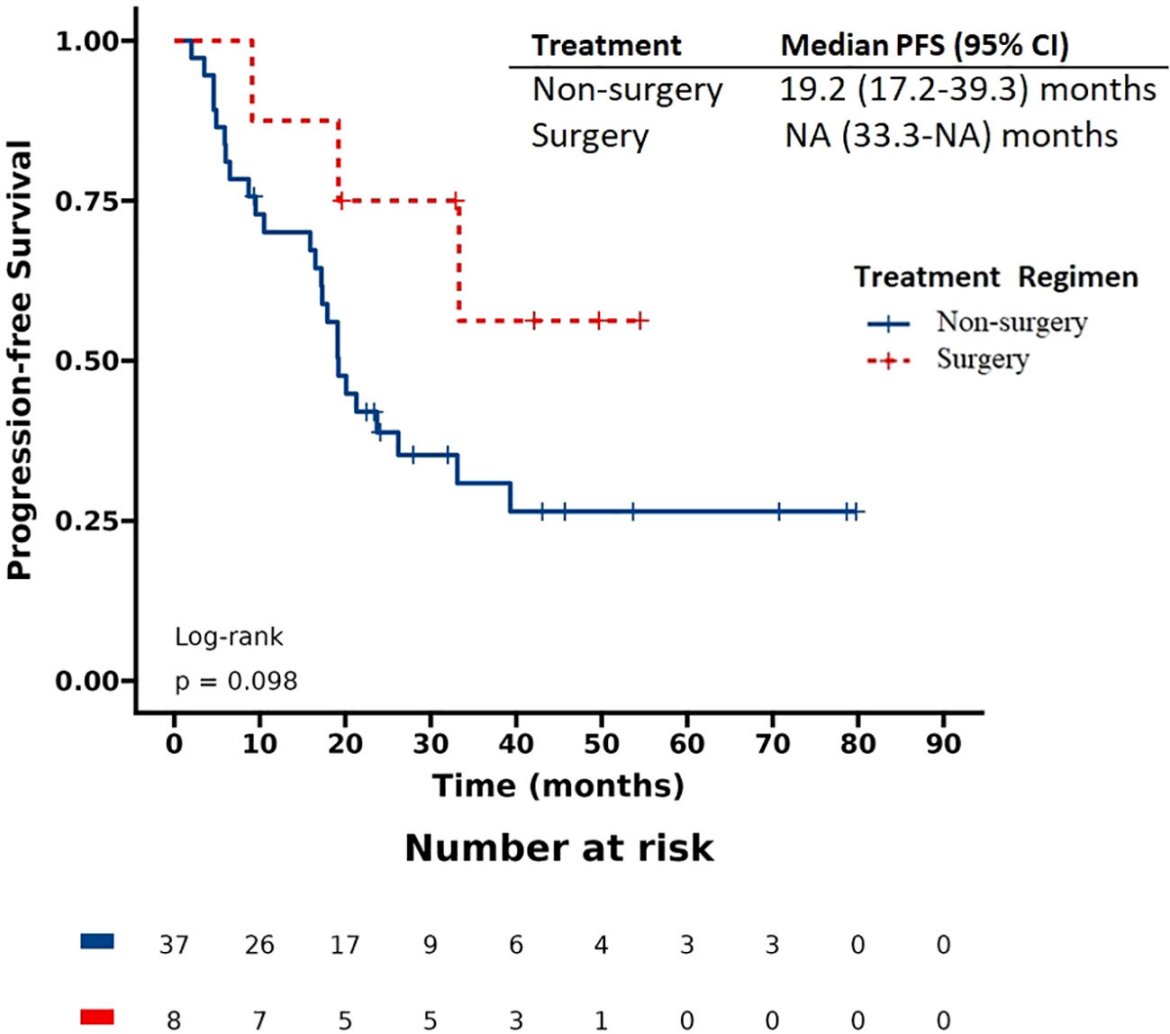
Progression-free survival (PFS) in Trastuzumab+Pertuzumab group.

**Figure 6:**
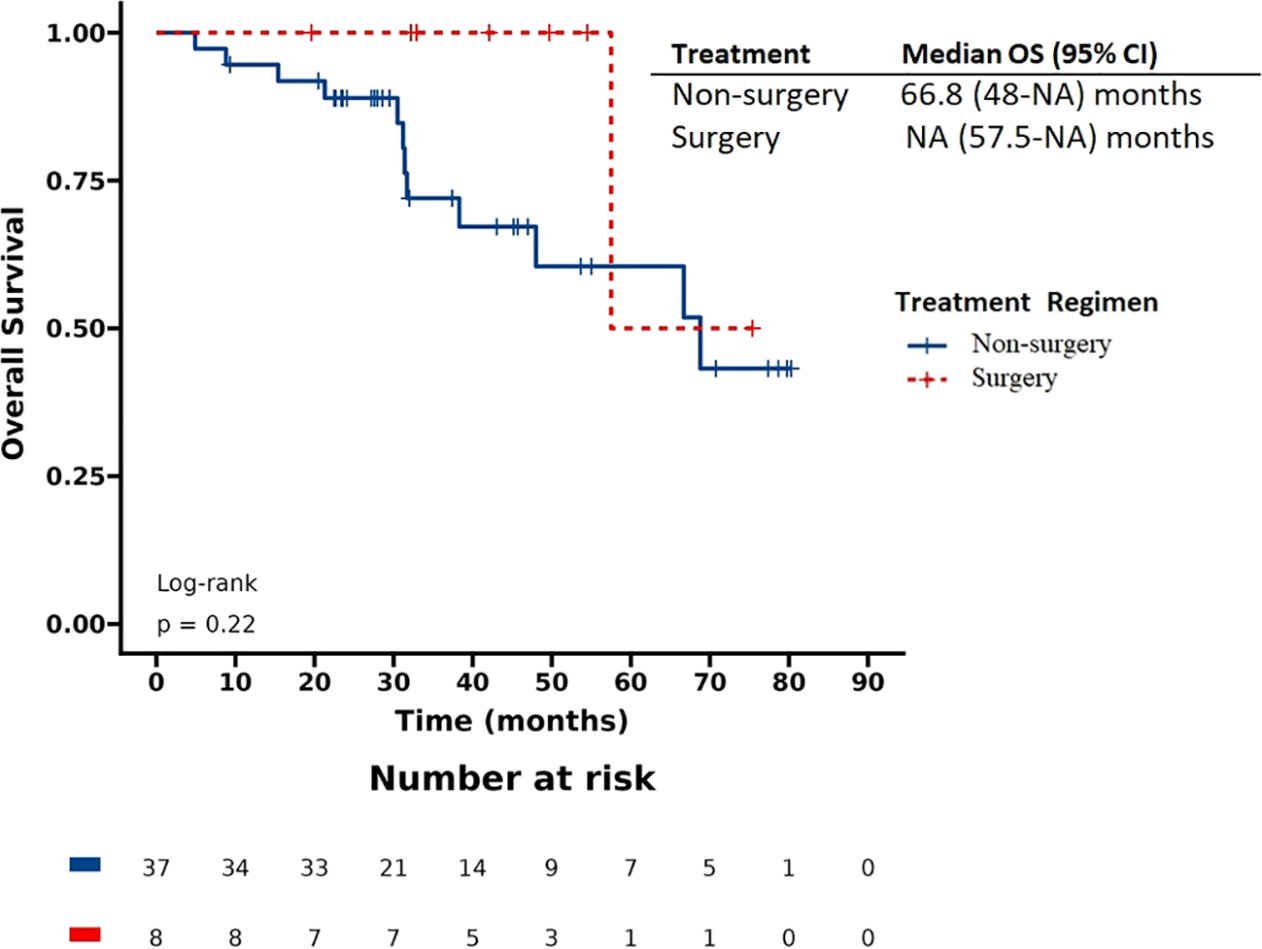
Overall survival (OS) in Trastuzumab+Pertuzumab group.

We conducted subgroup analyses to assess the potential benefits of surgery in different patient groups based on age, T stage, HR status, first-line target therapy, and tumor metastasis. Similarly, we did not find any subgroup that benefited from surgical treatment with PFS and OS (**Figures 3**, **4**). Additionally, we performed a survival analysis on patients who received first-line targeted therapy with trastuzumab plus pertuzumab. **Figures 5** and **6** present the results, indicating no statistically significant difference in the improvement of PFS and OS between patients who underwent surgery and those who did not.

## 4 Discussion

In our study, primary site surgery does not improve the prognosis of de novo stage IV HER2-positive breast cancer. De novo stage IV breast cancer refers to breast cancer that has already spread to other parts of the body at the time of diagnosis. Although there are currently no specific treatment guidelines regarding surgical intervention for patients with de novo metastatic breast cancer, several studies suggest that surgery may enhance survival rates for certain individuals (14, 15). It is currently believed that palliative topical treatment should only be given when the efficacy of systemic medication has stabilized or when local symptoms are severe. However, in clinical practice, approximately half of patients with advanced breast cancer receive surgical treatment (16).

The MF07-01 study, a prospective clinical trial conducted in Turkey, revealed that patients who received localized treatment exhibited better survival rates compared to those who did not (19% *vs*. 5% of 10-year survival). Further subgroup analyses indicated that individuals younger than 55 years, patients with only bone metastases, or those who tested positive for estrogen receptor (ER-positive) were more likely to benefit from localized treatment. This finding further establishes the importance of surgical treatment in current medical practice (7, 8). However, the results of the TATA study and the TBCRC013 study suggest that localized surgical management does not improve survival in patients with stage IV breast cancer (9, 10). The EA2108 phase III clinical trial, published in 2022, included patients who responded positively to systemic therapy. The study reported 3-year overall survival (OS) rates of 68.4% and 67.9% for the two groups, respectively. However, the difference between these rates was not statistically significant (17).

Studies utilizing large databases such as the Surveillance, Epidemiology, and End Results (SEER) and the National Cancer Database (NCDB) have demonstrated a significant improvement in overall survival rates associated with primary cancer surgery (18–20). Also, several retrospective studies have confirmed the effectiveness of surgical treatment in advanced breast cancer. Among these studies, it has been observed that HER2 positive patients are particularly responsive to surgical interventions (6, 13, 21, 22). However, the current clinical data lack currency, and the prognosis for patients in this category has improved considerably with the widespread use of anti-HER2 therapy. Therefore, it is necessary to further explore the role of local treatment in HER2-positive breast cancer in the era of new drug therapy.

In our study, we observed a trend towards improvement in progression-free survival (PFS) for patients who underwent localized surgery. However, this trend was not statistically significant, and there was no improvement in overall survival (OS) for these patients. In the multivariate Cox hazard model, it was found that patients who received Trastuzumab plus Pertuzumab as first-line systemic targeted therapy, rather than surgery, influenced both PFS and OS outcomes. No subgroup analysis suggested any benefit of surgical treatment for PFS and OS. Among patients with a low number of metastases and only bone metastases, as well as those with hormone receptor positive (HR+) tumors who typically have better survival rates, surgery did not show any profitability trends. This is possibly due to the fact that this subset of patients already had reasonable systemic lesion control through effective systemic therapy, negating the need for further local surgery to reduce tumor load. Nowadays, the first-line standard of treatment option for HER2-positive advanced breast cancer is Trastuzumab plus Pertuzumab. In our study, no benefit in terms of PFS and OS was found for surgical treatment in this population. However, it is important to note that some of the OS data in our study were not yet mature. Nevertheless, follow-up data indicated that some patients who underwent surgery experienced prolonged disease control and achieved the expected results.

Although it has been previously believed that surgical treatment can reduce tumor load and decrease the risk of tumor metastasis, thereby improving patient prognosis, particularly in patients with bone metastases only or oligometastases, Possible mechanisms for this effect include increasing the number of CD4 and CD8 T-cells to enhance the anti-tumor immune response, as well as removing some tumor stem cells to reduce the risk of metastasis (5, 23). However, our data suggests that surgical treatment should be evaluated in conjunction with systemic anti-tumor therapy. We have found that surgical treatment does not offer a prognostic benefit when systemic anti-tumor therapy is effective. The decision to undergo surgery for de novo stage IV HER2-positive cancer should be made on a case-by-case basis, considering factors such as the patient’s overall health, the extent of cancer, and the potential benefits and risks of surgery (5). No serious adverse events related to surgery were observed in this study. Our study demonstrated no significant correlation between the timing of surgical treatment and patient prognosis. We also aim to include a larger number of patients who have undergone surgical resection following systemic therapy, in order to evaluate the efficacy of systemic therapy prior to surgery and identify a subset of patients who may benefit from surgical treatment. Furthermore, due to the limited number of patients with stable metastases or complete response in our study, we were unable to analyze the prognosis of this specific subgroup. We hope that, in the future, we can establish guidelines for both local and systemic treatment regimens in this population, incorporating ctDNA and other tools.

Our study also has some limitations. Firstly, it should be noted that this was a retrospective study conducted at a single center, which inevitably introduces the possibility of selection bias. Therefore, the characteristics between the surgical and non-surgical groups were unbalanced. In addition, the sample size of this study is insufficient. And more and more patients with advanced breast cancer are receiving standard anti-tumor systemic therapy, and the proportion of surgical treatment is decreasing. The limited sample size caused some limitations to the statistical analysis. Additionally, the length of our follow-up period was not sufficient, which led to certain subgroups of patients not reaching the median survival time. This may have potentially affected the conclusions drawn from our study. Hence, it is important to consider future research findings with complete survival data.

## 5 Conclusion

In summary, our findings indicate that surgical treatment does not improve the prognosis of patients with HER2-positive primary advanced breast cancer. Given the current era of new drug development and the widespread use of anti-HER2 therapy to enhance patient outcomes, primary surgery is not recommended, except in exceptional circumstances.

## Data availability statement

The raw data supporting the conclusions of this article will be made available by the authors, without undue reservation.

## Ethics statement

The studies involving humans were approved by The Ethics Committee of the First Hospital of Jilin University. The studies were conducted in accordance with the local legislation and institutional requirements. The participants provided their written informed consent to participate in this study.

## Author contributions

GL: Conceptualization, Data curation, Formal Analysis, Writing – original draft, Writing – review & editing. LJ: Conceptualization, Funding acquisition, Methodology, Project administration, Resources, Writing – original draft, Writing – review & editing. RY: Data curation, Formal Analysis, Writing – original draft. YL: Data curation, Formal Analysis, Writing – original draft. ZL: Conceptualization, Methodology, Project administration, Resources, Writing – review & editing. JC: Conceptualization, Funding acquisition, Methodology, Project administration, Resources, Writing – review & editing.
